# B-cell depletion abrogates immune mediated cytopenia and rejection of cord blood transplantation in Hurler syndrome

**DOI:** 10.1038/s41409-021-01465-w

**Published:** 2021-10-04

**Authors:** R. Nataraj, P. Hiwarkar, D. Bonney, H. Campbell, S. Jones, D. Deambrosis, P. Evans, K. Poulton, P. M. van Hasselt, MB. Bierings, J. J. Boelens, C. A. Lindemans, R. Wynn

**Affiliations:** 1 Departments of Blood and Marrow Transplant, Royal Manchester Children’s Hospital, Manchester, M13 9WL UK; 2 Bai Jerbai Wadia Hospital for Children, Mumbai, India; 3 Center for Genomic Medicine, Saint Mary’s Hospital, Manchester, M13 9WL UK; 4 Paediatric Haematology and Oncology Department, Starship Children’s Hospital, Auckland, New Zealand; 5 Children’s Health Ireland at Crumlin, Dublin, Ireland; 6 Transplantation Laboratory, Royal Manchester Children’s Hospital, Manchester, UK; 7 Department of Paediatrics, Wilhelmina Kinderziekenhuis, UMC Utrecht, University Utrecht, Utrecht, The Netherlands; 8 Department of Blood and Marrow Transplant, Princess Maxima Center for Pediatric Oncology, Utrecht, The Netherlands; 9 Stem Cell Transplantation and Cellular Therapy, MSK Kids, Memorial Sloan Kettering Cancer Center, New York, NY USA

**Keywords:** Bone marrow transplantation, Transplant immunology

## Abstract

Umbilical cord blood is the preferred donor cell source for children with Inherited Metabolic disorders undergoing Hematopoietic Cell Transplant (HCT), and its use has been associated with improved “engrafted survival” and higher donor chimerism compared to other cell sources. However, as in other pediatric cord blood transplants for non-malignant disease, immune-mediated cytopenia and primary graft failure limit its use, and the latter remains the commonest cause of death following cord blood transplant for non-malignant disease. We have previously shown an association between immune-mediated cytopenia and graft failure in inherited metabolic diseases suggesting that both immune-mediated cytopenia and graft failure could be mediated by antibodies from the residual recipient B cells. Since rituximab is effective in depletion of B cells and management of refractory immune-mediated cytopenia following HCT, we have added rituximab to the conditioning regimen. We studied 57 patients in 2 centers who received myeloablative conditioning for cord blood transplant in Hurler syndrome, and report a significant improvement in event-free survival with reduced incidence of graft failure and without any evidence of immune-mediated cytopenia in those patients that had received rituximab.

## Introduction

Hematopoietic stem cell transplantation (HCT) is the preferred treatment for several lysosomal storage diseases (LSD), including Hurler Syndrome (HS) [1]. Use of umbilical cord blood (UCB) is preferred due to advantages such as reduced time between diagnosis and transplant, better tolerance of HLA-mismatch, reduced chronic Graft versus Host Disease (GvHD), higher chimerism and enzyme in engrafted recipients with improved disease-related outcomes [1–5].

However, the advantages of cord blood transplantation are eclipsed by increased incidence of graft failure (GF) and immune-mediated cytopenia (IMC) in LSD [2, 6]. GF is relatively common in children receiving UCB HCT for non-malignant disease (NMD) and was indeed the commonest cause of death in a large multi-institutional review of such transplants in children [7]. The cause of such increased GF is multifactorial and includes that the cell dose might be limiting, that the patients are more immune competent and better able to reject the graft than heavily immune-suppressed leukemic children, and that they are sometimes sensitized by blood products prior to conditioning. IMC are also more common after CB transplant in non-malignant disease (NMD) [8, 9]. We recently reported the clinical and serological features of IMC following CB transplant in HS and suggested that IMC is a *forme fruste* of graft rejection, in which a residual intact host immune system rejects the cellular products of the engrafted CB, and which is sometimes associated with subsequent progression to aplastic graft failure [10]. In this report we had not identified a contribution of these other factors such as HLA match, conditioning and cell dose to the occurrence of GF. We identified antibodies in the recipient against antigens that were present on cord cells but not host cells which were consistent with allo-rejection of cord cells by residual intact host immunity, but which did not exclude auto-rejection by engrafted cord immune cells.

Discordance in the depletion of circulating and tissue lymphoid cells in a non-human primate model after myeloablative conditioning is described [11]. Tissue lymphoid cells persist even after complete depletion of circulating lymphoid cells following conditioning. We therefore hypothesized that B-cell depletion with rituximab might attenuate the antibody responses contributing to GF and IMC that are mediated by recipient B cells resident in the lymphoid organs.

To deplete and prevent the recovery of recipient B cells, we added 2 doses of rituximab, at days −10 and +30 respectively, to myeloablative conditioning in children receiving CBT for HS. We report the effect of this B-cell depletion with rituximab on the incidence of IMC and GF in the first cohort of patients from 2 transplant centers.

## Materials and methods

This study was a retrospective analysis of consecutive patients undergoing first UCB HCT for HS at the Royal Manchester Children’s Hospital, United Kingdom (2010–2020) and the University Medical Center in Utrecht, The Netherlands (2009–2020). All patients received a standard conditioning regimen with Busulfan (area under the curve, 90 mg/h/L), fludarabine (160 mg/m2), and serotherapy with Anti-thymocyte globulin (ATG), Thymoglobuline (5–10 mg/kg) [12, 13]. From 2019, Rituximab 375 mg/m2 was added as part of conditioning, as above.

Transplant demographic details and events are recorded in Table 1. Variables recorded and analyzed were conditioning drugs, graft-versus-host disease (GVHD) prophylaxis, pre-conditioning, and day 0 absolute lymphocyte count (ALC), HLA mismatch, age at transplant, gender, total nucleated cell dose, GVHD (grade 1 or higher), chimerism and B lymphocyte count post HCT.

**Table 1:** Patient characteristics.

	Control group (*n* = 37)	R group (*n* = 20)	*P* value
Male	17 (45.9%)	8 (40 %)	
Median age at HSCT	14 months (IQR- 7,18.4)	8.6 months (IQR- 4.9, 18.9)	0.17
HLA mismatch			0.08
0 (HLA matched)	23	4	
1 HLA Locus mismatch	8	10	
2 HLA loci mismatch	3	4	
>2 HLA Loci mismatch	3	2	
Total nucleated cell count (Median)	14.5 * 10^7^/kg	16.2 * 10^7^/kg	0.67
Absolute Lymphocyte count prior to HSCT (Median)	5.8 * 10^9^/L	5.3 *10^9^/L	0.36
Absolute Lymphocyte count at Day zero (Median)	0.03 * 10^9^/L	0.01 * 10^9^/L	0.20
Neutrophil engraftment (Mean)	Day 17.1 days	Day 16.6 days	0.7
GVHD			0.53
Acute GvHD, Grade 1–2	15	8	
Acute GvHD, Grade 3–4	6	1	
Chimerism (Mean)	97.9 %	94.7 %	0.07

For each patient IMC, GF, and Death were recorded. IMC included autoimmune hemolytic anemia (AIHA), autoimmune thrombocytopenia (AIT) and autoimmune neutropenia (AIN). AIHA was defined by a positive direct agglutinin test (DAT), testing for both IgG and C3d detection, and/or a rapid decrease in erythrocytes combined with markers of hemolysis such as an increased reticulocyte level, elevated serum bilirubin, reduced plasma haptoglobin, and raised serum lactic dehydrogenase. AIT and AIN were defined by a rapid decrease in platelets (<100 * 109/L) and neutrophils (1.0 *109/L), respectively, with or without detection of antibodies against platelets and neutrophils and when other causes of cytopenia were excluded [10]. In cases in which a specificity of the red blood cell antibody could be identified, red blood cell genotyping of donor and recipient was then performed. If IgG was detected and there was hemolysis but no specificity against RBC could be determined, it was classified as “aspecific antibodies.”

Graft failure was sub-categorized into primary or secondary, and into aplastic or with autologous reconstitution [2]. Primary aplasia was defined as no neutrophil engraftment by day +42 and primary autologous reconstitution as neutrophil engrafted by day +42 but with <20% donor-derived hematopoiesis. Secondary aplasia defined as sustained cytopenias following neutrophil engraftment with fully donor-derived hematopoiesis (⩾95%) and secondary autologous reconstitution as falling donor chimerism (to <20%) following neutrophil engraftment and adequate donor-derived hematopoiesis [2]. Both IMC and GF were grouped in immune events. Event free survival (EFS) was defined as IMC-free GF-free survival following the first HCT. Overall survival (OS) was defined as survival from first HCT to last follow-up or death.

Categorical variables have been summarized as number and percentage, continuous variables as median and range. Categorical outcomes between the two groups were compared by Chi-square test (Fisher exact for expected count <5). Continuous and ordinal outcomes between the two groups were compared by Mann–Whitney U test. We also compared time dependent outcomes by survival analysis using Kaplan–Meir curves and Log rank test. *P* value of <0.05 was considered significant. Statistical analyses were performed with Statistical Package for Social Sciences, statistical software package (version 25.0, IBM).

## Results

Fifty-seven patients who underwent first UCB transplant for HS with median age of 12 months (IQR- 6, 18.7 months) were included. Transplant demographic details are recoded in Table 1.

**Fig. 1:**
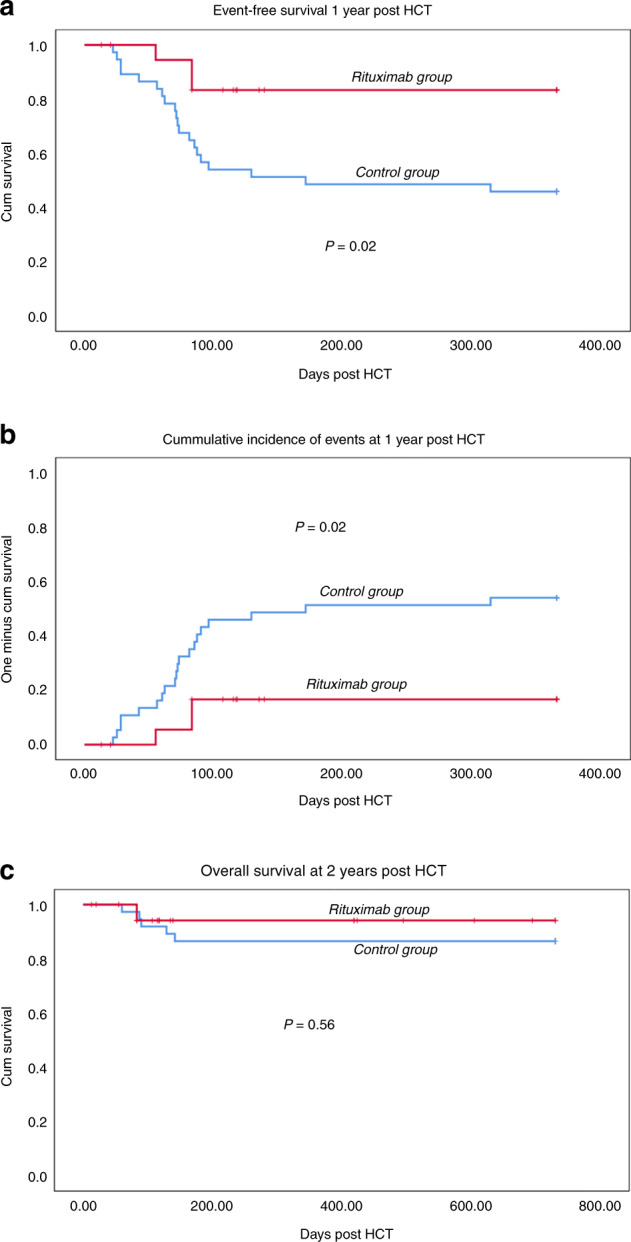
The event-free survival at 1-year post HCT in patients receiving myelo-ablative conditioning for MPSIH. The events are IMC, GF and death, and there are significantly reduced events, one death only, in those that have received rituximab. The overall survival at 2-years post HCT is not different between those that have received rituximab in conditioning therapy and those that have not, since the death events are not significantly different between the 2 groups and the “immune events” are usually rescued with medical intervention or second transplants. **a** Event-free Survival at 1-year post HCT (Events are Immune-mediated cytopenia, Graft failure and death). **b** Cumulative incidence of immune events at 1 year post HSCT. **c** Overall survival at 2-years post HCT.

Twenty patients received rituximab in their conditioning protocol and 37 did not. Thirteen patients developed IMC at a median time of 72 days (IQR- 59, 90) post HCT, none in the rituximab group (Tables 2 and 3). Where red cell antibodies were detected then their specificity was such that they were consistent with allo-antibodies, made by a residual intact host immune system against cord blood red cells, as previously described [10]. All three cell lines were affected in 4 patients [8, 10]. GF was observed in 6 patients in total and median time was 28 days post HCT (IQR- 26.5, 101 days) which included one patient from the rituximab group. When we compared the occurrence of events between two groups (IMC, GF and/or death) there was a significant difference as depicted in Table 2 and Fig. 1. Event free survival (EFS) at 1 year-post HCT was 85% and 45.9% in the rituximab group and control group respectively (*p* = 0.02) (Fig. 1a, b). Overall, 6 patients died, 1 from the rituximab group. The main causes of death were sepsis with multiorgan failure, adenoviral disease, and cardiomyopathy. Overall survival at 2 years post HCT was not different among the two groups (95 % vs 86.5%, *p* = 0.56) (Fig. 1c), since both IMC and GF were rescued with medical interventions and second transplant, respectively. All 6 patients with GF underwent 2nd HCT with cord blood in 3, matched unrelated donor in 2 and Matched sibling donor in 1.

**Table 2:** Incidence of immune events (IMC, GF, and Death).

	Control group (*n* = 37)	Rituximab group (*n* = 20)
Immune- mediated cytopenia	13 (35%)	0
Autoimmune hemolytic anemia	10/13	
Autoimmune thrombocytopenia	10/13	
Autoimmune neutropenia	5/13	
Median time	72 days (IQR- 59, 90)	
Graft failure	5 (13.5%)	1 (5%)
Primary Graft failure	3/5	1
Secondary Graft failure	2/5	
Median time	28 days (IQR- 26.5, 101 days)	30 days
Death	5 (13.5%)	1 (5%)

**Table 3:** Antibodies identified in patients with Immune-mediated cytopenia.

Serial Number	IMC	DAT	Antibodies	Identified antibodies
Patient 1	AIHA	IgG+; C3+	Not reported	Not reported
Patient 2	AIHA, AIN, AIT	IgG+; C3+	Positive	Anti-e antibodies^*^
Patient 3	AIHA, AIN, AIT	IgG+; C3+	Positive	Anti-e antibodies^*^
Patient 4	AIT, AIN	Weakly positive	Not reported	Not reported
Patient 5	AIT	C3d+	Positive	Pan autoantibodies
Patient 6	AIHA	IgG+; C3+	Positive	Anti E / Anti Kell antibodies^*^
Patient 7	AIHA	IgG+; C3+	Positive	Anti-JkA antibodies^*^
Patient 8	AIHA, AIN, AIT	IgG+; C3+	Positive	Anti E, Anti c antibodies^*^Pan autoantibodies
Patient 9	AIHA, AIN, AIT	IgG+; C3+	Positive	Warm, aspecific autoantibodies, IgM
Patient 10	AIT	Positive	Not reported	Not reported
Patient 11	AIHA, AIT	IgG+; C3+	Positive	Anti-e antibodies
Patient 12	AIHA, AIN, AIT	IgG+; C3+	Positive	aspecific auto antibodies, autoantibodies against thrombocytes and granulocytes
Patient 13	AIHA, AIT	IgG+; C3+	Positive	warm and cold aspecific auto Antibodies

*Antibody specificity consistent with either allo-antibody (recipient directed at cord red cells) or autoantibody.

*AIHA* auto-immune hemolytic anemia, *AIN* autoimmune neutropenia, *AIT* autoimmune thrombocytopenia.

**Fig. 2:**
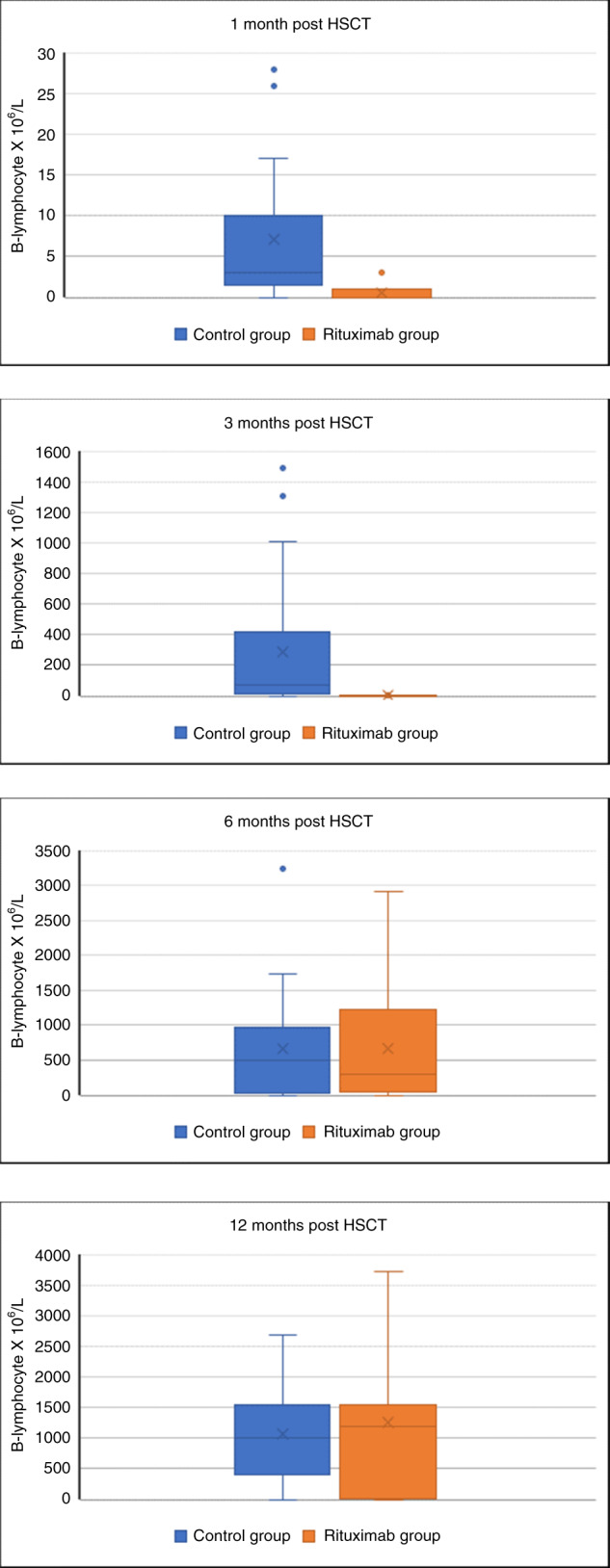
Box-and-whisker plots comparing B lymphocyte recovery at 1, 3, 6, and 12 months post HCT. The B cell count is significantly reduced at 1 and 3 months after HCT, but is fully recovered by 9 months. Immunoglobulin substitution was routinely given until B cell recovery. The Box-and-Whisker plot indicates the median value (center line), the 25th–75th percentiles (box), and the 10th–90th percentiles (whiskers) at various time-points after HCT in both groups.

We compared B lymphocyte recovery at 1, 3, 6, and 12 months post HCT as depicted in Table 4 and Fig. 2. B-cell recovery was delayed in the rituximab cohort compared to the control group at one and three months after HCT, but was not statistically different than the control group by 6 months, and was fully recovered by 9 months. There was no difference in the incidence of infection between the 2 groups. Immunoglobulin substitution was routinely given until B-cell recovery and IgM production.

**Table 4:** B lymphocyte count post HCT.

	Control group (*n* = 37)	Rituximab group (*n* = 20)	*P* value
	Median (* 10^6^ /L)	Median (* 10^6^ /L)	
1 month	3	0	0.00
3 months	177	0	0.00
6 months	488	125	0.89
9 months	879	1445	0.65

## Discussion

Both GF and IMC are features of UCB transplant, especially in those with NMD, and GF is the commonest cause of death in pediatric UCB transplant recipients for NMD [7]. The cumulative incidence of IMC in inherited metabolic diseases is reported to be 22% from various studies and with median onset of about 4–6 months [14]. We have reported a temporal change in the pattern of graft failure (GF) in LSD transplant. Previously such GF was characterized by late autologous reconstitution, and this was eliminated with pharmacokinetic monitoring to deliver increased, myeloablative doses of busulfan. In recent years, GF has principally been aplastic, particularly common in CB recipients, and is considered a manifestation of a failure of recipient immunoablation [2, 6]. We have also reported progression of IMC to secondary GF, and pre-conditioning lymphocyte count and absolute lymphocyte count on day 0 as predictors of IMC [10]. We have therefore postulated that both GF and IMC are related manifestations of immune-mediated rejection of cord stem cells or mature cells derived from the engrafted cord. Both are manifestations of a failure of recipient immune suppression and are commoner in NMD than leukemia since the latter have received immunosuppressive chemotherapy prior to transplant [7]. The presence of antibodies in IMC and in GF has led us to postulate the involvement of the humoral immune system in these phenomena, that significantly attenuate the utility of cord blood HCT in children with NMD.

We report that the B-cell depletion with rituximab abrogates immune-mediated cytopenia and rejection of cord blood transplantation in Hurler syndrome, and propose that impact of addition of rituximab to the conditioning regimen for UCB transplant in children with other NMD should be assessed. The exact basis for this observed therapeutic response to rituximab lies either in the removal of auto-reactive cord-derived B cells or of allo-reactive recipient B cells until competent and properly regulated donor-derived immunity is engrafted.
